# Systematic Evaluation of Reference Protein Normalization in Proteomic Experiments

**DOI:** 10.3389/fpls.2013.00025

**Published:** 2013-02-27

**Authors:** Henrik Zauber, Vivian Schüler, Waltraud Schulze

**Affiliations:** ^1^Max Planck Institute of Molecular Plant PhysiologyGolm, Germany; ^2^Plant Systems Biology, University of HohenheimStuttgart, Germany

**Keywords:** mass spectrometry based proteomics, label-free proteomics, absolute quantitation, protein spiking, data normalization

## Abstract

Quantitative comparative analyses of protein abundances using peptide ion intensities and their modifications have become a widely used technique in studying various biological questions. In the past years, several methods for quantitative proteomics were established using stable-isotope labeling and label-free approaches. We systematically evaluated the application of reference protein normalization (RPN) for proteomic experiments using a high mass accuracy LC-MS/MS platform. In RPN all sample peptide intensities were normalized to an average protein intensity of a spiked reference protein. The main advantage of this method is that it avoids fraction of total based relative analysis of proteomic data, which is often very much dependent on sample complexity. We could show that reference protein ion intensity sums are sufficiently reproducible to ensure a reliable normalization. We validated the RPN strategy by analyzing changes in protein abundances induced by nutrient starvation in *Arabidopsis thaliana*. Beyond that, we provide a principle guideline for determining optimal combination of sample protein and reference protein load on individual LC-MS/MS systems.

## 1 Introduction

In modern large-scale experiments involving high throughput omics-data, proper normalization strategies are required to allow for meaningful comparison of different replicated sample runs and experiments. Starting from spectral counting methods (Ishihama et al., 2005; Lu et al., 2007) proteomic mass spectrometry is moving toward analyzing peptide ion intensities for analyzing protein abundances since efficient software for quantification of proteomic raw data became available (Cox and Mann, 2008; Mortensen et al., 2010; Pedrioli, 2010; Specht et al., 2011). While spectral counts can give an estimation even of absolute protein abundances, dynamic range as well as statistical power of calculated protein abundances is higher by averaging protein specific peptide ion intensities (peptide intensities; Aebersold and Mann, 2003; Steen and Mann, 2004; Colinge and Bennett, 2007; Choudhary and Mann, 2010; Arike et al., 2012). Normalization and quantitation of peptide intensities can be done by introducing isotopically labeled peptides to a sample for which full scan spectra will be co-analyzed (Ong et al., 2002). The heavy and light peptide forms can be separated by their mass and one of the isotope species serves as a reference in quantitation (reference peptide; Arsova et al., 2012a,b). If supplied at known concentrations, the isotope-labeled reference peptides can also be used for determination of absolute protein concentrations (Kirkpatrick et al., 2005; Hanke et al., 2008). Beside stable-isotope labeling, label-free quantitation strategies for peptide intensity analysis are becoming very popular due to their easy and inexpensive experimental designs. However, this usually comes at the cost of accuracy (Arsova et al., 2012b) and a data analysis procedure which is mainly based on relative changes of ion intensity fractions between different treatments (Zauber and Schulze, 2012). Absolute quantitation without stable-isotope labeling is also possible by employment of protein abundance indices from spectral counting methods such as emPAI (Ishihama et al., 2005) or APEX (Lu et al., 2007). The intensity based absolute quantitation (iBAQ; Schwanhäusser et al., 2011) uses a spiked mixture of unlabeled reference proteins as a basis for conversion of emPAI values to proteome-wide calculate protein concentrations (Schaab et al., 2012). In a comparison of these label-free absolute quantitation strategies, iBAQ turned out to produce the least variation and also corresponded well to biochemical total protein quantification (Arike et al., 2012). Spiked reference proteins were used for absolute quantification in MS experiments making use of the observation that the average of the three most intense tryptic peptides of a protein correlates to its protein concentration, independent of peptide sequence (Silva et al., 2006). Thus, Reference Protein Normalization (RPN) is a simple method for sample normalization and quantification without strong impacts on overall sample complexity. Advantages particularly lie in an easy experimental design and sample preparation, without being limited to analysis of relative comparisons between protein intensity fractions. The RPN technique is based on addition of a protein of known concentration as a reference point for normalization of sample peptide intensities. Here, we systematically explored the potential of RPN in a complex plant protein background by using bovine serum albumin (BSA) as a reference protein. The plant proteome, at least when working with green tissue, contains proteins with a highly skewed abundance distribution due to the over-representation of the carbon fixing protein Ribulose-1,5-bisphosphate Carboxylase Oxygenase (Rubisco). Therefore, dynamic range and type of normalization has great influence on quantitative data analysis. For validation of the RPN strategy we analyzed protein intensity changes in response to nutrient starvation.

## 2 Results

### 2.1 Relationship between peptide intensities and spiked BSA amounts

**Figure 1:**
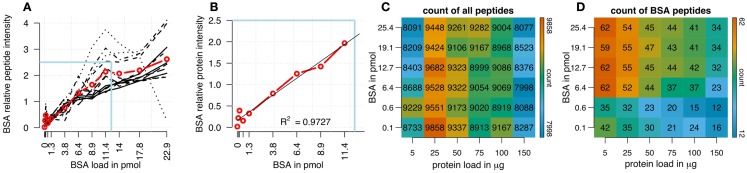
**(A)** Ion intensities of BSA peptides expressed as fraction of total sample ion intensity. BSA intensities normalized on fraction of total ion intensity sums were proportional to the supplied amounts of BSA. Linearity of averaged intensities (red) was observed for BSA amounts up to 12.8 pmol. Variation between relative intensities of different peptides increased for BSA loadings above 5 pmol. **(B)** Enlargement of boxed region in **(A)** showing linear relationship between the averaged relative ion intensities of BSA peptides and BSA concentrations (*R*^2^ = 0.97). Count of all identified plant and BSA peptides is shown, in a matrix combining different amounts of BSA loads (120 fmol to 25.6 pmol) with different total protein loadings (5–150 μg). **(C)** Count of identified plant peptides was dependent on sample complexity with an optimum at 25 μg of protein load. The amount of spiked BSA amount had almost no impact on identified plant peptides. **(D)** Count of BSA peptides was affected by total protein load as well as BSA load. Optimal combinations were 5 and 25 μg of total protein spiked with 1–4 pmol of BSA. The color bar indicates the color code for low and high counts of peptides from blue to red.

We analyzed the impact of different amounts of spiked protein concentrations of bovine serum albumin (BSA) on BSA peptide intensities in a complex sample background (Figures 1A,B). In a first analysis using cRacker (Zauber and Schulze, 2012) the fraction of total ion intensity sum normalization was applied and eight BSA peptides were identified and quantified in all samples. When expressed as fraction of total ion intensity sums, BSA peptide ion intensities were observed to be proportional to the spiked amount of BSA (Figure 1A). Saturation of most BSA peptide ion intensities occurred at BSA spikes of more than 12 pmol. The variation between individual BSA peptide ion intensities increased at BSA loadings above 6–9 pmol. A linear relation between protein intensity and spiked BSA amounts was observed in the range of 0.6–11 pmol of BSA (Figure 1B).

### 2.2 Influence of different total protein background

**Figure 2:**
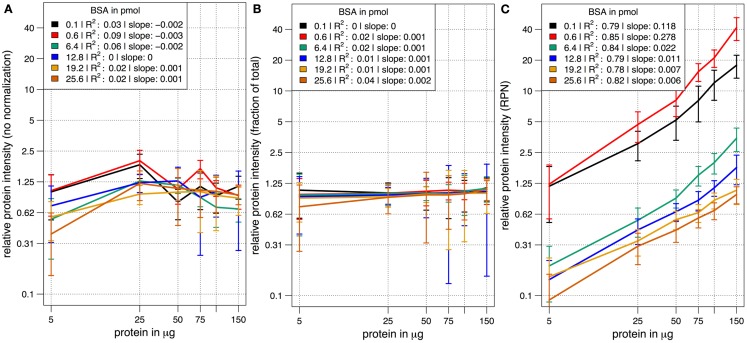
**The median scaled protein intensity sum of all identified proteins at different total protein loads and different amounts of spiked BSA**. Results from three different normalization methods are shown in double logarithmic scale plots. **(A)** Median curve of non-normalized, but scaled protein intensities followed no specific pattern. **(B)** Normalization on total ion intensity sum per sample did not display differences in total protein amount at the different protein loadings. Instead, the average abundance fraction is constant throughout. **(C)** With RPN a linear relationship of protein intensities with increased amounts of protein became apparent at all combinations of total protein and spiked BSA.

**Figure A1:**
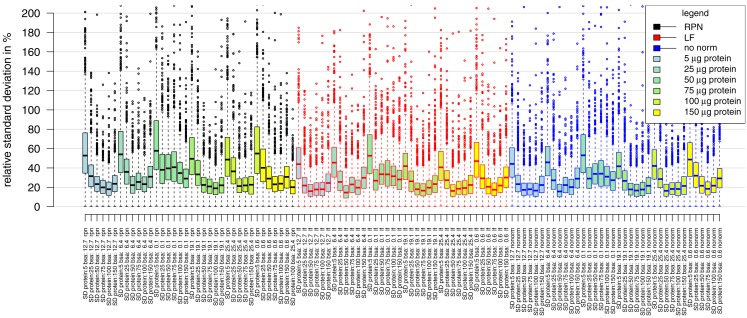
**Boxplots presenting distributions of relative standard deviations of median averaged protein intensities**. Analyzed samples belong to the combinatorial matrix spiked with BSA (from 0.1 pmol to 25 pmol) in a protein background ranging from 5 μg to 150 μg. Distribution of standard deviation is comparable between the different type of normalizations. Rpn, reference protein normalization; if, label free normalization based on fraction of total peptide ion sum; nonorm, no normalization was applied.

In a combinatorial matrix we systematically analyzed the influence of spiked amount of BSA (from 0.1 to 25 pmol) on the total number of identified plant peptides. Total protein amounts ranged from 5 to 150 μg (Figures 1C,D). With the given LC-MS/MS setup, it became apparent that the amount of spiked BSA had almost no impact on the total number of identified plant peptides (Figure 1C). However, count of identified plant peptides was dependent on sample complexity with an optimum between 25 and 50 μg of protein load (see also raw ion intensity values in Data Sheet S1 in Supplementary Material). This is in agreement with previously published data (Arsova et al., 2012b). Not surprisingly, highest counts of BSA peptides could be detected in the two samples with the lowest total protein background (Figure 1D) and more BSA peptides were detected with higher amounts of spiked BSA. Without normalization, but after scaling of peptide ion intensities across samples, the median curve of BSA protein intensity sums was independent of protein or BSA load and showed a tendency for highest ion intensities at 25 μg of total protein (Figure 2A). Normalization of all identified protein intensity sums to total sample ion intensity compensated differences between ion intensities at different total protein loads. Differences in total protein were not visible, since only the relative abundance fractions were compared (Figure 2B). With RPN, a direct proportionality between normalized protein intensity sum and the actual total protein amount could be extracted from the data (RPN normalized protein intensities are available in Data Sheet S2 in Supplementary Material). The slope of this proportionality decreased with higher spiked amounts of BSA and was highest with 600 fmol of BSA. The relative standard deviations (expressed as coefficient of variation: standard deviation divided through averaged protein intensity) of RPN were on average below 25% when a total protein amount of 50 μg spiked with 600 fmol of BSA was used. In general, relative standard deviations of RPN were comparable to standard label-free normalization (Figure A1 in Appendix; Figure 1). RPN was also tested for its compatibility with using spectral counting. Therefore emPAI (Ishihama et al., 2005) values from the cRacker analysis (Zauber and Schulze, 2012) were normalized on emPAI values of the reference protein BSA. The relationship between spiked BSA amounts and sample protein emPAI values was best for the two lowest spiked BSA amounts. In general, variance was much higher with the coefficient of determination ranging only from 0.14 to 0.5 compared to when using ion intensity values. Spiking of BSA amounts higher than 6.4 pmol seemed to be already exceed the dynamic range (Figure A1 in Appendix; Figure 2). In conclusion, RPN quantitation applied on peptide ion intensities outperforms RPN on spectral counting and should be chosen due to higher precision and dynamic range in protein quantitation.

### 2.3 Validation of RPN in the context of nutrient starvation

**Figure 3:**
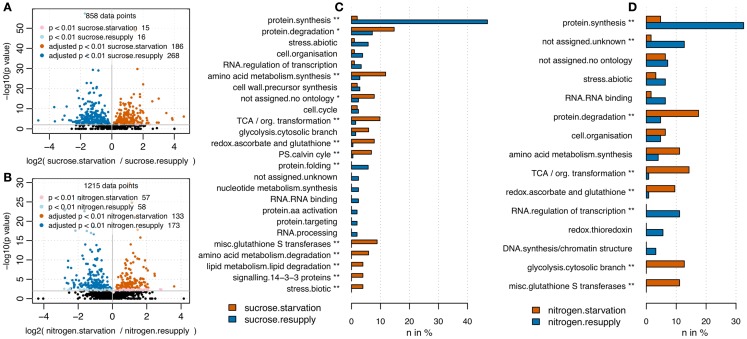
**Volcano plots comparing full nutrition condition with starvation for sucrose (A) and nitrogen (B)**. P values have been derived from pairwise t-test analysis using log_2_ transformed peptide intensities. Multiple testing correction was applied (Benjamini and Hochberg, 1995). Upon sucrose starvation, 244 proteins were up-regulated and 342 proteins were down-regulated. Upon nitrogen starvation, 135 proteins were up-regulated and 177 down-regulated. Barplots visualize functional categories based on MapMan bins (Usadel et al., 2005) being significantly up-regulated (red) or down-regulated (blue) upon nutrient starvation. Only bins being represented by more than two proteins are shown. For sucrose **(C)** as well as nitrogen starvation **(D)** the bin “protein.synthesis” was significantly depleted under starvation, whereas the bins “glycolysis.cytosolic branch,” “misc.glutathione S transferases,” “redox.ascorbate and glutathione,” “TCA/org. transformation” were significantly up-regulated under full nutrition. In general starvation under sucrose affected slightly more categories (11 significantly overrepresented bins) compared to nitrogen starvation (8 significantly overrepresented bins). *Uncorrected p value < 0.05; **Benjamini Hochberg corrected p value < 0.05.

RPN was applied to quantify changes in protein abundance induced by nitrogen or sucrose starvation. Following the results from the optimization of total protein and BSA spike combinations, we used 35 μg of total protein spiked with 3.8 pmol of BSA. The rather high amount of BSA was chosen to ensure identification of a large number of BSA peptides across all samples, but still was without drastic impact on total count of peptide identifications (Figure 1C). Upon sucrose starvation, 186 proteins were up-regulated and 268 proteins were down-regulated (pairwise t-test p < 0.01 after multiple testing correction; Benjamini and Hochberg, 1995) compared to full nutrition (Figure 3A). Upon nitrogen starvation the same analysis revealed 133 significantly up-regulated and 173 down-regulated proteins (Figure 3B). Results from the nutrient starvation experiment are summarized in Data Sheet S3 in Supplementary Material. The differentially expressed proteins were mapped to MapMan (Usadel et al., 2005) functional categories and tested for over-representation using a Fisher Exact test (uncorrected p values < 0.05). Under both starvation conditions, protein synthesis functions were significantly down-regulated, while protein degradation was up-regulated (Figures 3C,D; Brouquisse et al., 1992). A general significant nutrient starvation response under both starvation conditions affected up-regulation of TCA cycle, amino acid synthesis, and glycolysis.

### 2.4 Validation of RPN applicability for absolute quantitation

**Figure 4:**
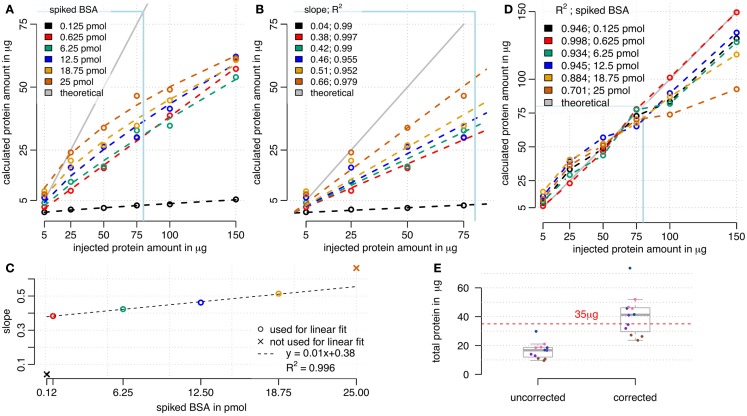
**Calculation of absolute protein amounts by RPN**. **(A)** Sum of calculated absolute protein amounts in comparison to injected total protein amount. Saturation effects occurred with high total proteins amounts and high BSA spikes, while lower BSA spikes up to 6 pmol showed a linear relationship. **(B)** A window from 5 to 75 μg of protein load was used for linear modeling, with setting 0 as an intercept. **(C)** The slope of the relationship between calculated and injected total protein amount depended on spiked BSA amount. **(D)** Corrected sum of calculated absolute protein amounts by multiplying with reciprocal slope values from **(B)**. Multiplication of cumulated absolute protein loads with corresponding reciprocal slopes **(D)** improved approximation to ideal curve especially for lower BSA loads **(C)**. **(E)** Cumulated absolute protein amounts of the starvation experiments were underestimated using 3.8 pmol of BSA and 35 μg of protein, but approximated the injected protein amount after correction. Technical replicates are indicated by equal colors. The correction factor was calculated using the linear model from **(D)**.

We used RPN for calculating absolute protein amounts by only using the three most intense peptide ions of a protein for quantitation as described (Silva et al., 2006). Correlations between the calculated sum of protein amounts and the injected total protein amounts were compared (Figure 4A). For all BSA spike concentrations used, a relationship between calculated and injected protein amount was visible. However, there was a tendency for under-estimation of injected protein amount, particularly at high total protein injections. With increasing reference protein concentrations, saturation effects of this relationship were observed. Spiked BSA amounts below 6.25 pmol resulted in the largest linear approximation. As expected, higher BSA loads seem to compete with sample protein for column binding, explaining the increased under-estimation of loaded protein at high total protein amounts. A correction factor was obtained from linear fits using concentration window from 5 to 75 μg of protein (Figures 4B,C). A linear correlation between obtained slopes and injected BSA amounts was found for spiked BSA ranging from 0.625 to 18.75 pmol (Figure 4D). We decided to use a linear fit mainly for practical reasons, even though a non-linear fit may achieve even better performance. By multiplying sums of calculated protein amounts (Figure 4A) with corresponding inverse slopes (Figure 4B) resulted in a better approximation of calculated protein amounts to the expected values (Figure 4C). Correction worked best for BSA loadings between 0.625 and 6.25 pmol in the range of 5–75 μg of protein load (Figure 4C). In contrast, high BSA spikes (12.5–25 pmol) at lower amounts of protein (5–25 μg) were overestimated and high protein amounts (100–150 μg) remained underestimated. Therefore the selected amounts of protein (35 μg) and BSA (3.8 pmol) used in the starvation experiments (Figure 3) are in a range that is suitable for this correction model. We tested this model on the starvation experiments and calculated a BSA load specific correction factor (Figure 4E). Results show that variation between biological samples was higher than for technical replicates. Since technical replicates were created after tryptic digest by equally splitting the samples, we conclude that the analytical workflow is highly reproducible. Therefore, the bigger impact on technical variation was related to steps before tryptic digest. In general, the distribution of calculated total protein amounts showed that the uncorrected values were underestimated, while most of the corrected values well approximated the original injected protein amounts (Figure 4E).

## 3 Discussion

### 3.1 BSA as an internal reference standard

With modern LC-MS/MS instrumentation in combination with suitable data analysis, peptide intensities can be reproducibly and precisely quantified. Using a reference protein based normalization, averaged proteins displayed a linear relationship with the loaded amounts of reference protein until detector saturation was reached (Figure 1A). This finding is the basis for using a single protein as an internal standard for normalization and determination of absolute protein amounts. Different labs use instrumental setups that can vary in protein loading capacity and dynamic and linear range of the mass spectrometer. Therefore it is necessary to systematically explore the best combination of sample total protein and reference protein amount. For the optimal use of RPN it is important to choose a reference protein concentration that is within the linear range of the detector. This will ensure precise normalization and quantitation across a wide range of peptides. Loading above 12 pmol of BSA on a nano-flow-HPLC coupled with a LTQ-Orbitrap resulted in increased variation between individual peptides as some peptides reached saturation of ion intensities. Furthermore, it could be shown that the proportionality between reference protein normalized ion intensity and loaded protein amount was independent of spiked amounts of BSA. However, slopes from the correlation analysis between amounts of spiked BSA and sample protein varied and this influenced dynamic range for quantitation. Therefore, the optimal spike concentration should allow a fairly high number of peptide identifications of the reference protein as well as high numbers of total peptide ion identifications. Based on these criteria, we chose to use 25–75 μg of total protein spiked with 1–4 pmol of BSA. Other studies used BSA loads in a range from 0.2 pmol of BSA (Chang et al., 2012) up to 30 pmol (Wu et al., 2006). The BSA amounts used here were within this range and were selected to minimize effects from BSA spiking on total number of sample peptide identifications while still giving sufficient and reproducible identifications of BSA peptides. As reference protein, we generally recommend to use a protein from a foreign species to ensure that normalization does not interfere with co-analyzed sample peptides. However, a variant of the RPN strategy is based on a sample internal reference protein (iRPN). This internal reference protein would have to follow similar criteria as “house-keeping” reference genes in qRT-PCR or western blots. For plants, at least on the basis of transcripts, condition-dependent reference genes have been published (Czechowski et al., 2005). An advantage of iRPN over RPN is that it keeps the technical error low, as additional sample preparation steps in using RPN can increase the error. Therefore in RPN, total sample protein as well as BSA concentration and spiking of BSA, each needs to be adjusted and handled accurately. Systematic errors between samples can be compensated using RPN. In contrast to that, the error introduced by biological variation of the reference protein using iRPN is hard to control. Therefore the challenging part of iRPN lies in finding a protein with a reliable stable expression across all experimental samples. A meaningful application of iRPN would be in proteomic analysis of co-immunoprecipitations using the prey as the internal sample reference. RPN is in general suitable for untargeted analysis of high and low complex protein mixtures. If necessary, RPN would even allow calculating rough estimates of absolute protein amounts by only considering the three most intense peptide ions, which were shown to correlate with protein abundance independently of the chemical nature of these peptides (Silva et al., 2006). While with RPN a direct correlation between calculated cumulative protein amounts and injected total protein amount could be observed, column saturation effects and under-estimation of absolute protein with increasing total protein amounts are limiting this technique. Therefore, it is necessary to work below maximum column binding capacity and to define a correction factor for the applied reference protein concentration (see Figures 4C,D). This correction factor turned out to be mainly dependent on the reference protein concentration, when working in the linear range window (Figure 4A). An optimal load of BSA for absolute quantitation was found to be between 0.625 and 6.25 pmol of BSA. However, absolute quantification using RPN is likely to give only a rough estimation of absolute protein amounts. For experiments that do not require absolute quantitation, we recommend to include scaling of peptide intensities across all samples without limiting the analysis to only the three most intense peptides per protein. This ensures a sufficiently large basis for statistical analysis when combining peptide ion intensities to protein ion intensity sums.

### 3.2 Application of RPN in comparative proteomics

We used alterations in protein abundances upon nutrient starvations as an illustration for the potential of RPN to analyze biological processes. Sucrose and nitrogen are two important nutrients for plant growth and development (Nicolaï et al., 2006; Rolland et al., 2006; Osuna et al., 2007; Wind et al., 2010; Eveland and Jackson, 2012). The two main nutritional functions of sucrose are energy and carbon supply, providing necessary carbon skeletons for nitrogen assimilation (Wang et al., 2003; Scheible et al., 2004). Nitrogen is important for many synthesis pathways. It is component of amino acids and nucleotides which are building blocks for protein and nucleic acids synthesis. Additionally sucrose as well as nitrogen in the form of nitrate also serve as signaling components triggering cell wide responses (Brouquisse et al., 1991; Aubert et al., 1996; Wang et al., 2000, 2012; Contento et al., 2004; Scheible et al., 2004; Lee et al., 2007; Gutiérrez, 2012). These cellular responses are manifested as alterations in protein abundances and their post-translational modification status (Niittylä et al., 2007; Engelsberger and Schulze, 2012). Using RPN for the analysis of prolonged sucrose and nitrogen starvation-induced protein abundance changes, we could track several well known proteome-wide responses (Figures 3C,D). Protein synthesis was drastically depleted under both starvation conditions. While this pathway was decreased under sucrose starvation as a response to reduced energy availability, under nitrogen starvation this also resulted from a decreased availability of amino acids as building blocks for protein synthesis. The increased abundance of proteins with functions in protein degradation under starvation conditions might be an indication for an increased recycling of nitrogen and carbon from degraded proteins. In support of this, it is known that the starvation-induced up-regulation of TCA cycle proteins, besides functional role in NADH production, can also serve as supply of precursors for biosynthesis of several amino acids (Fernie et al., 2004). Under nitrogen starvation, when amino acid synthesis is limited and protein synthesis is drastically decreased (Richard-Molard et al., 2008), the excess of carbon skeletons needs to be metabolized either through respiration, indicated by significant increase of “TCA/org. transformation” and “glycolysis.cytosolic branch,” or by increased starch synthesis (Wang et al., 2003), indicated by significant up-regulation of starch synthesis enzymes such as phosphoglucomutase or sucrose phosphate synthase under nitrogen starvation (Data Sheet S2 in Supplementary Material). Under sucrose starvation, cells need to reduce energy consuming biosynthesis pathways and to mobilize alternative energy suppliers like lipids (Aubert et al., 1996) or other sugar species derived from non-starch polymers (Lee et al., 2007). In our experiments, we found that especially lipids (“lipid metabolism.lipid degradation”) have been mobilized and degraded to provide alternative energy sources.

### 3.3 Conclusion

We could show that reference protein normalization can be applied to complex proteomic datasets and its application results in biologically meaningful data. The addition of a reference protein to a sample protein mixture does not interfere with the analysis of total plant protein if used in an optimal combination. RPN can in principle be applied to any kind of sample after joint digestion of reference protein and total protein extract. When using RPN for the first time on a given instrumental setup it is recommended to test for optimal combinations of the reference protein and total protein load. Once the optimal combinations are found, they can be applied in principle to many different experiments over a long time until the system setup is changed drastically, such as the usage of different type of column material, doubled column length or changed types of instruments. The optimal combination found here can serve as a starting point also of such optimizations. Particularly for lower complexity samples such as gel slices, RPN could outperform normalization on total ion intensity sums since they are very much dependent on sample complexity. RPN is not designed to overcome metabolic or isotopic labeling. Moreover, it is simple and inexpensive alternative in label-free analysis to avoid percentage based comparisons as it could even result in information on absolute protein concentration (Silva et al., 2006). Therefore, the strength of this method lies in application to systems and model plants that are not readily accessible for large-scale metabolic labeling.

## 4 Methods

### 4.1 Cell suspension culturing and nutrient depletion experiments

*Arabidopsis* cell suspension cultures were grown in full mineral JPL medium and subcultured in fresh medium every week (Jouanneau and Péaud-Lenoël, 1967). Cultures were harvested for protein extraction after 5 days of growth in fresh medium. For comparison of protein amounts under different nutritional conditions, cultures grown on full medium were subcultured either to sucrose-depleted medium or nitrogen-depleted medium for 2 days. Control cultures were subcultured to full nutrition medium for 2 days.

### 4.2 Sample preparation

After harvesting suspension cell cultures with vacuum funnel, cells were frozen in liquid nitrogen and then ground by mortar and pestle. Protein was extracted from powdered material using an extraction buffer [50 mM TrisHCl pH 7,5; 20% (w/v) Glycerol; 1% PVPP; 5 mM DTT]. After pelleting of cell debris, the supernatant was subjected to chloroform/methanol extraction to isolate soluble proteins. Precipitated protein pellets were resuspended in 8 M urea, 2 M thiourea. Protein concentration was determined using Bradford assay (Bradford, 1976). The stock solution of BSA was prepared in 2 mM Tris buffer pH 8. The final BSA concentration was adjusted to 260 μM and confirmed by using NanoOrange Protein Quantitation Kit (Invitrogen). BSA volumes with defined amounts of solubilized protein were spiked into each sample after adjusting total protein content to the desired amount. Relative volumes of BSA where thereby kept below 10% of total sample volume, to prevent dilution effects in protein digest. For optimization of total protein and BSA amount combinations, mixtures of different amounts of total plant protein and BSA were prepared before tryptic digestion. BSA amount was varied between 125 fmol and 25 pmol, while total protein amount was varied between 5 and 150 μg.

### 4.3 Sample preparation for proteomic analysis

Protein in 6 M urea, 2 M thiourea, pH 8 were reduced, carbamidomethylated (Sechi and Chait, 1998) and directly digested with LysC (3 h) at room temperature. After diluting the sample solution by four volumes, using 2 mM Tris pH 8, trypsin was added for over night digest at room temperature (Olsen et al., 2004; Kierszniowska et al., 2009). The digest was stopped by adding trifluoroacetic acid to reach a pH of around 2. Tryptic peptides were desalted over C18 Stop And Go Extraction tips (Empore Disk, Varian, Inc.; Rappsilber et al., 2003).

### 4.4 LC-MS/MS analysis and protein identification

Injections, ranging from 25 to 150 μg of protein, were analyzed by LC-MS/MS using nano-flow HPLC (Proxeon Biosystems) and an Orbitrap hybrid mass spectrometer (LTQ-Orbitrap XL, Thermo Scientific) as mass analyzer. Peptides were eluted from a 75 μm analytical column (Reprosil C18, Dr. Maisch GmbH) on a linear gradient, running from 5 to 80% acetonitrile in 90 min at a flow rate of 250 nl/min. Up to five data-dependent MS/MS spectra were acquired in the linear ion trap for each FTMS full scan spectrum acquired at 60,000 full-width half-maximum resolution settings with an overall cycle time of approximately 1 s. Raw file peak extraction, protein identification, and quantitation of peptides was done with MaxQuant (version 1.2.2.5) using a protein sequence database of *Arabidopsis thaliana* (TAIR10, 35,386 entries, www.arabidopsis.org). For protein identification, carbamidomethylation, and N-terminal protein acetylation were used as fixed modifications and methionine oxidation as a variable modification. Standard settings in MaxQuant involving peptide false discovery rate of 0.01, minimum peptide length of six amino acids and enabled retention time correlation, with a time window of 2 min were used. Mass accuracy was set to 6 ppm for full scans and 0.5 Da for MS/MS scans.

### 4.5 Quantitation and reference protein normalization

Peptide lists derived from MaxQuant (evidence.txt) were directed to cRacker (Zauber and Schulze, 2012) analysis for normalization between samples and for merging peptide intensities to protein intensities. Peptides which were not quantified in less than 50% of all samples were filtered out. RPN normalization is implemented within cRacker and this option was used for the data analysis. cRacker specific parameters and settings are provided as Data Sheet S4 in Supplementary Material. The principal steps of the reference protein normalization were: (1) BSA peptides present in all samples were selectively median scaled across all samples. Resulting scaled BSA peptide intensities were median averaged to sample specific BSA protein intensities. (2) All peptide intensities within each sample were normalized on the calculated BSA protein intensity from step 1. (3) Peptide intensities with missing values in more than half of all samples were filtered out. (4) Remaining normalized peptide intensities were median scaled and median averaged. Resulting RPN normalized protein intensities were directed to statistical analysis.

### 4.6 Absolute quantitation using RPN

For absolute quantitation of protein intensities the three most intense peptides were mean averaged and normalized on the resulting BSA intensity value of each sample using cRacker. The options “top3” and “reference protein normalization” were selected. All peptides were included in the analysis. The absolute protein amounts could be calculated by referring each protein intensity to co-measured reference protein intensities of known amounts of substance. To indicate precision of this method, amount of substance was converted to mass when analyzing approximation of summed protein weights to used total protein weight. Molecular weights were calculated using “Compute pI/Mw tool” web tool from ExPASy (Wilkins et al., 1999). Linear fit of the response curves was done in R (Team, 2009) using the function lm, forcing intercept point at 0. Correction of total protein weight was done by multiplying calculated absolute protein amounts with the reciprocal slope values.

### 4.7 Statistics

Protein abundance changes upon alteration of nutritional status were tested by pairwise t-test on log_2_ transformed intensities of normalized peptides (normalized on reference protein BSA). Multiple testing correction was applied according to Benjamini Hochberg (Benjamini and Hochberg, 1995). Significant proteins (p < 0.01) were mapped to MapMan functional categories (Usadel et al., 2005). Over representations of functional categories were determined using a Fishers Exact test (*α* = 0.05).

### 4.8 Protocol for evaluating RPN

1. Extract enough protein (around 0.5–1 mg of protein) to be used as the same sample background in different LC-MS/MS runs.
2. Inject increasing amount of total protein extract in the range between 1 and 100 μg in order to define injected protein amount giving highest number of peptide identifications. The injected protein amount resulting in highest peptide/protein identification will be used as the optimal total protein load.
3. Spike increasing amounts of BSA or any other reference protein, that is not related to the species used in the complex sample, into complex protein mixtures by using the optimal protein load determined in step 2. We recommend reference protein amounts from 100 fmol up to 20 pmol.
4. Identify and quantify the raw data after LC-MS/MS measurements. Use fraction of total normalization and check median intensities of the BSA peptides. We recommend to only use ion intensities of peptides identified in all samples. After plotting fraction of total sum BSA protein intensity against amounts of loaded BSA one can identify the linear range of ion intensity quantitation on the given mass spectrometer (compare Figure 1A). For RPN it is necessary to spike with a BSA amount, that lies within this linear range. In addition it needs to be checked that the total number of identified peptides in the complex sample is not strongly affected by the addition of the reference protein.
5. Combine information from optimal protein load (step 2) and from optimal BSA load (step 4) to conclude the optimal combination of total protein and reference protein in using RPN on another LC-MS system.

## Conflict of Interest Statement

The authors declare that the research was conducted in the absence of any commercial or financial relationships that could be construed as a potential conflict of interest.

## Supplementary Material

The Supplementary Material for this article can be found online at http://www.frontiersin.org/Plant_Proteomics/10.3389/fpls.2013.00025/abstract

Supplementary Data Sheet S1 **Intensity values of all peptides identified in the combinatorial matrix**.

Supplementary Data Sheet S2 **RPN normalized and median averaged protein intensities from combinatorial matrix**.

Supplementary Data Sheet S3 **Results from nitrogen and sucrose starvation experiments**.

Supplementary Data Sheet S4 **Parameter files that can be loaded into cRacker in order to use same settings as used here**.

## Figures and Tables

**Figure A2:**
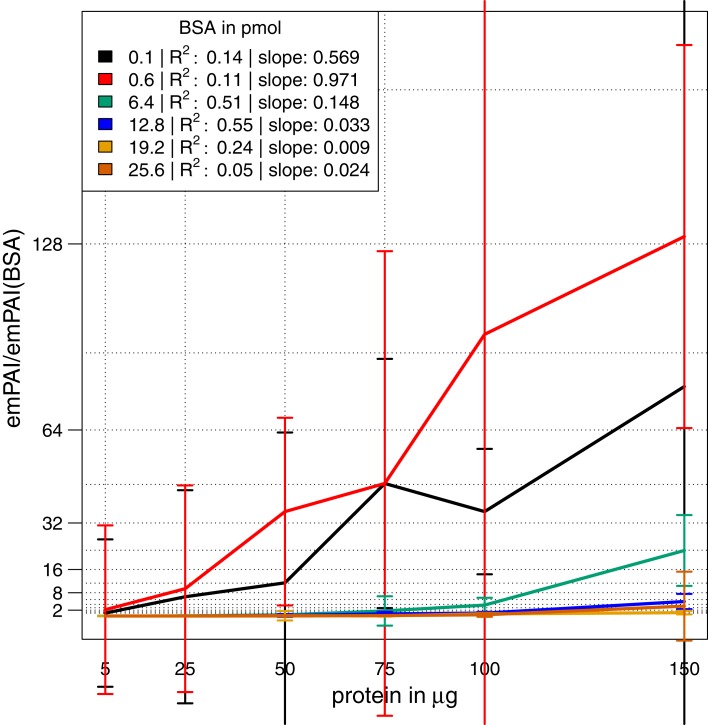
**The use of RPN in quantitation based on spectral counting**. emPAI values of reference protein BSA and the sample proteins were used as quantitative readout. In general, standard deviations were higher compared to RPN based on peptide ion intensity values.
